# Prenatal features and neonatal management of severe hyperparathyroidism caused by the heterozygous inactivating calcium-sensing receptor variant, Arg185Gln: A case report and review of the literature

**DOI:** 10.1016/j.bonr.2021.101097

**Published:** 2021-06-09

**Authors:** Marion Aubert-Mucca, Charlotte Dubucs, Marion Groussolles, Julie Vial, Edouard Le Guillou, Valerie Porquet-Bordes, Eric Pasmant, Jean-Pierre Salles, Thomas Edouard

**Affiliations:** a Department of Medical Genetics, Toulouse University Hospital, Toulouse, France; b Endocrine, Bone Diseases, and Genetics Unit, Reference Center for Rare Diseases of Calcium and Phosphate Metabolism, ERN BOND, OSCAR Network, Children's Hospital, Toulouse University Hospital, Toulouse, France; c Department of Obstetrics and Gynecology, Toulouse University Hospital, Toulouse, France; d Department of Radiology, Children's University Hospital, Toulouse, France; e Department of Genetic and Molecular Biology, Cochin Hospital, AP-HP Centre-Université de Paris, Paris, France

**Keywords:** Calcimimetics, Calcium-sensing receptor, Familial hypocalciuric hypercalcemia, Neonatal severe hyperparathyroidism

## Abstract

**Background:**

Loss-of-function variants in the calcium-sensing receptor (*CASR*) gene are known to be involved in a clinical spectrum ranging from asymptomatic familial hypocalciuric hypercalcemia (FHH) to neonatal severe hyperparathyroidism (NSHPT). Homozygous or compound heterozygous variants are usually responsible for severe neonatal forms, whereas heterozygous variants cause benign forms. One recurrent pathogenic variant, p.Arg185Gln, has been reported in both forms, in a heterozygous state. This variant can be a *de novo* occurrence or can be inherited from a father with FHH.

NSHPT leads to global hypotonia, failure to thrive, typical X-ray anomalies (diffuse demineralization, fractures, metaphyseal irregularities), and acute respiratory distress which can be fatal. Phosphocalcic markers show severe hypercalcemia, abnormal urinary calcium resorption, and hyperparathyroidism as major signs.

Classical treatment involves calcium restriction, hyperhydration, and bisphosphonates. Unfortunately, the disease often leads to parathyroidectomy. Recently, calcimimetics have been used with variable efficacy. Efficacy in NSHPT seems to be particularly dependent on *CASR* genotype.

**Case presentation:**

We describe the antenatal presentation of a male with short ribs, initially suspected having skeletal ciliopathy. At birth, he presented with NSHPT linked to the pathogenic heterozygous *CASR* variant, Arg185Gln, inherited from his father who had FHH. Postnatal therapy with cinacalcet was successful.

**Discussion:**

An exhaustive literature review permits a comparison with all reported cases of Arg185Gln and to hypothesize that cinacalcet efficacy depends on *CASR* genotype. This confirms the importance of pedigree and parental history in antenatal short rib presentation and questions the feasibility of phosphocalcic exploration during pregnancy or prenatal *CASR* gene sequencing in the presence of specific clinical signs. It could in fact enable early calcimimetic treatment which might be effective in the *CASR* variant Arg185Gln.

## 1 Background

The calcium-sensing receptor (CASR), a G-protein-coupled receptor mainly expressed in parathyroid glands and kidneys, acts as a key regulator of calcium homeostasis (Hofer and Brown, 2003). Under normal conditions, the CASR is activated in response to high extracellular calcium concentrations which leads to parathyroid hormone (PTH) secretion inhibition by the parathyroid cells and inhibition of calcium reabsorption in renal tubule cells. However, lower than set-point calcium concentrations lead to CASR inactivation which triggers PTH secretion and renal calcium reabsorption (Pollak et al., 1993). Loss-of-function variants in the *CASR* gene [MIM* 601199] alter the set point for activation, thereby decreasing CASR sensitivity to calcium concentration. These inactivating variants are involved in a wide clinical spectrum ranging from benign and often asymptomatic familial hypocalciuric hypercalcemia (FHH; MIM#145980) to severe neonatal hyperparathyroidism (NSHPT; MIM#239200) (Marx and Sinaii, 2020). Heterozygous loss-of-function *CASR* variants usually lead to FHH, whereas homozygous or compound heterozygous *CASR* variants result in NSHPT (Marx and Goltzman, 2019). However, pathogenic heterozygous loss-of-function *CASR* variants have also been described in some cases of NSHPT, notably the c.554G>A p.(Arg185Gln) missense pathogenic variant (Fisher et al., 2015; Reh et al., 2011; Forman et al., 2019; Gannon et al., 2014; Obermannova et al., 2009; Bai et al., 1997).

NSHPT is usually diagnosed during the first weeks of life in the presence of signs of severe hypercalcemia and hyperparathyroidism including poor feeding, polyuria, failure to thrive, hypotonia, respiratory distress caused by thoracic restriction, and fractures (Forman et al., 2019). Typical biochemical features include hypercalcemia and hypophosphatemia related to hyperparathyroidism, and low fractional excretion of urinary calcium (Marx and Goltzman, 2019). Bone X-ray abnormalities include diffuse demineralization, metaphyseal irregularities, cortical dualization, subperiosteal erosion, and fractures consistent with hyperparathyroidism (Marx and Sinaii, 2020; Roizen and Levine, 2012). If severe hypercalcemia in NSHPT is not detected and treated early, it can lead to potentially life-threatening complications or neurodevelopmental sequelae (Wilhelm-Bals et al., 2012).

Medical treatment of NSHPT is usually based on a combination of calcium restriction, hyperhydration, and bisphosphonates (Sun et al., 2018). When medical treatment fails, the only effective therapy consists of surgical parathyroidectomy with or without autotransplantation of parathyroid tissue in the forearm. However, this surgery can be insufficient and is associated with postoperative complications, especially in infants (Forman et al., 2019). A few case reports have described the efficacy of calcimimetics (cinacalcet), which are allosteric CASR agonists, in some forms of NSHPT (Sun et al., 2018).

In this paper, we describe the prenatal bone and renal features and postnatal management of a new case of NSHPT caused by the pathogenic heterozygous inactivating *CASR* variant Arg185Gln. We compared the changes in our patient under cinacalcet therapy with those in published case reports.

## 2 Case report

### 2.1 Case presentation

**Fig. 1:**
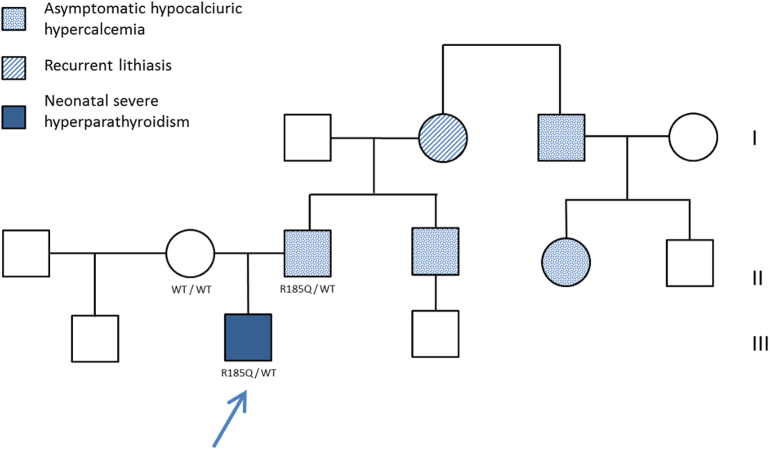
Pre- and postnatal features of NSHPT and changes under treatment. a. Fetal computed tomography at 26 weeks of gestation showing short ribs (see arrows). b. Postnatal skeletal survey showing narrowed thoracic cage with short ribs and multiple rib fractures (see arrows). c. Skeletal X-rays at 6 months of age, showing complete resorption of rib damage under cinacalcet therapy. d. Fetal computed tomography at 26 weeks of gestation showing irregular femoral, tibial and fibula metaphyseal ends (see arrows). e. Postnatal skeletal survey showing diffuse osteopenia with coarse trabecular markings, subperiosteal bone resorption, cortical dualization and metaphyseal corner fractures (see arrows). f. Skeletal X-rays at 6 months of age, showing complete resorption of long bone damage with cinacalcet therapy.

A healthy 41-year-old woman was referred to our center after the 22-week gestational ultrasound revealed short ribs and a possible craniosynostosis in the fetus on the second trimester ultrasound. After detailed genetic counselling, an amniocentesis was performed at 24 weeks to investigate the etiology. Array CGH was normal without any unbalanced chromosomal rearrangement, and so were *FGFR2* and *FGFR3* recurrent variant screening (to rule out *FGFR* related craniosynostosis syndromes), and the 7-dehydrocholesterol level (to rule out Smith-Lemli-Opitz syndrome). At 26 weeks, computed tomography confirmed short ribs with irregular ends but no craniosynostosis and overall renal cortex echogenicity was noted on ultrasound (Fig. 1). The association of bone and renal abnormalities led to an initial diagnosis of a skeletal ciliopathy spectrum disorder such as Jeune syndrome (asphyxiating thoracic dystrophy). Pregnancy was then complicated by hydramnios requiring amniotic fluid drainage at 33 weeks, which triggered fetal bradycardia and the need for a caesarean delivery. At birth, the new-born male measured in the low normal range for gestational age with a weight of 1800 g (32nd centile), length of 43 cm (39th centile), and an occipitofrontal circumference of 30 cm (26th centile) without craniosynostosis. Soon after birth, he developed hypotonia and respiratory distress requiring oxygen and non-invasive ventilation. Except for a bell-shaped chest, the rest of his clinical examination was unremarkable. A chest X-ray showed a narrowed thoracic cage with short ribs and multiple rib fractures. Subsequently, a skeletal survey revealed diffuse osteopenia with coarse trabecular markings, subperiosteal bone resorption, cortical dualization and metaphyseal corner fractures (Fig. 1). The initial laboratory evaluation revealed severe hypercalcemia (ionized calcemia: 1.66 mmol/l; reference range: 1.17–1.27), a slightly low phosphate level (1 mmol/l; reference range: 1–1.95), normal alkaline phosphatase levels (387 IU/l; reference range: 122–469), abnormal urinary calcium (calcium-to-creatinine ratio: 0.78 mmol/l; reference range: 0.2–2.0), and an increased PTH level (325 pg/ml; reference range: 15–65). The diagnosis of NSHPT was then suspected and confirmed by a phosphocalcic NGS panel which revealed the pathogenic heterozygous (PM1, PM2, PM5, PP2, PP3, PP5) variant c.554G>A p.(Arg185Gln) in the *CASR* gene (NM_000388.3).

**Fig. 2:**
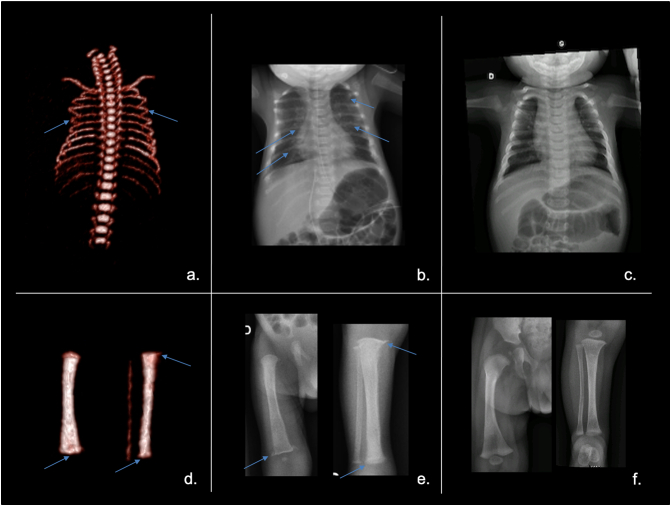
Familial pedigree of the proband (indicated by an arrow) Dotted line: individuals with asymptomatic hypocalciuric hypercalcemia; hatch fill: individuals with recurrent lithiasis; solid fill: individual with severe neonatal hyperparathyroidism. *CASR* genotype reported under the proband and his parents.

Calcium metabolism tests and genetic screening were then requested from both asymptomatic parents. These analyses revealed hypercalcemia (total serum calcium: 3.32 mmol/l, reference range: 2.2–2.6 mmol/l), low phosphate levels (0.59 mmol/l, reference range: 0.84–1.4 mmol/l), low calcium-to-creatinine ratio (0.16 mmol/mmol, reference range: 0.2–0.6 mmol/mmol) and hyperparathyroidism (PTH: 42 pg/ml, reference range: 15–65) in the father who harbored the same heterozygous *CASR* variant. Mineral homeostasis (25-hydroxyvitamin D level: 24 ng/ml) and *CASR* sequencing were normal in the mother. The family history revealed that the paternal grandmother also had FHH discovered as a result of recurrent urinary lithiasis (Fig. 2). Similarly, FHH affected various members of the paternal branch.

### 2.2 Treatment

**Fig. 3:**
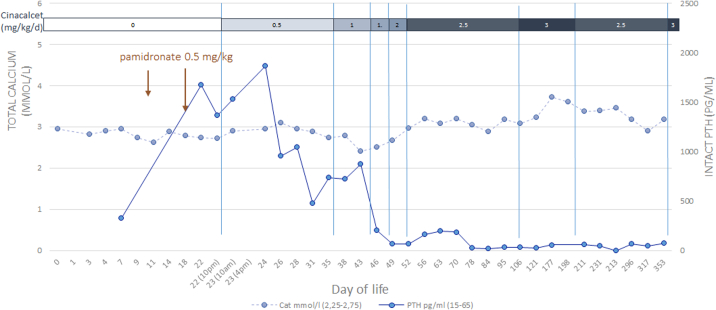
Changes in biochemical parameters under cinacalcet treatment.

Initial therapy included hyperhydration, phosphate supplementation and a low-calcium milk formula. Hypercalcemia did not improve. Therefore, treatment with pamidronate (0.5 mg/kg intravenous on days 9 and 14) was started. After a moderate transient response to pamidronate, serum calcium levels subsequently increased and were associated with very high PTH levels (1671 pg/ml). Clinically, the patient had persistent restrictive lung disease caused by significant rib fractures requiring oxygen and analgesics. Therefore, after confirmation of the genetic diagnosis of NSHPT treatment with calcimimetics (cinacalcet) was initiated on day 22 at 0.5 mg/kg PO daily and progressively increased to 3 mg/kg in 2 doses. The cinacalcet dose titration normalized the PTH in 25 days but serum calcium remained at approximately 3 mmol/l (Fig. 3).

### 2.3 Follow-up and outcomes

Hyperparathyroidism control provided significant improvement in clinical signs. The patient was discharged on day 73, and oxygen therapy could be discontinued at 6 months of age. Psychomotor development and growth were normal. At 6 months of age, X-rays showed complete normalization of bone abnormalities (Fig. 1) and ultrasound revealed nephrocalcinosis. At 11 months of age, parathyroid gland ultrasound showed no abnormality.

## 3 Material and methods

DNA extraction was performed with the Maxwell 16 LEV Blood DNA Kit (Promega, Charbonnières-les-Bains, France) on an EDTA blood sample. Experiments were performed at the NGS facility at Cochin Hospital, Paris (Assistance Publique-Hôpitaux de Paris AP-HP, France). A customized hybridization panel (*Roche NimbleGen*, Madison, WI, USA) and a NextSeq 500 system (Illumina, San Diego, CA, USA) were used to sequence the coding and IVS flanking (25 bp) regions of eight genes associated with parathyroid disorders (*AIP*, *AP2S1*, *CASR CDC73*, *CDKN1B*, *GCM2*, *GNA11*, *MEN1*). After demultiplexing and generation of FASTQ files, the sequence analysis was performed according to the Genome Analysis Tool Kit (GATK) guidelines using the Polyquery (Université de Paris, France) and MOABI (AP-HP) bio-informatic platforms. Variant pathogenicity was assessed according to the American College of Medical Genetics and Genomics and the Association for Molecular Pathology (ACMG-AMP) guidelines (Richards et al., 2015).

We performed an exhaustive review of the literature using the PubMed database to compile clinical data on individuals with the same pathogenic variant, Arg185Gln in NSHPT and all descriptions of cinacalcet therapy in NSHPT (Table 1) (Pollak et al., 1993; Fisher et al., 2015; Reh et al., 2011; Forman et al., 2019; Gannon et al., 2014; Obermannova et al., 2009; Bai et al., 1997; Szalat et al., 2015; Heath et al., 1996) using the following terms: “NSHPT”, “neonatal severe hyperparathyroidism”, “R185Q”, “p.(Arg185Gln)”, “*CASR*”, “CaSr”, “CaSeR”, “cinacalcet”, and “calcimimetics”.

**Table 1:** Reports of clinical presentation and changes in NSHPT with the pathogenic heterozygous *CASR* variant Arg185Gln under cinacalcet therapy.

References	Inheritance	Gender	Prenatal features	Postnatal features	Narrowed thorax	Nephro-calcinosis	X-rays description	Pamidronate	Cinacalcet	
									Age at start	Dose at normal PTH
Current report	Paternal inheritance	M	Ribs and renal abnormalities, hydramnios	Initial respiratory distress at birth, narrowed thorax	Yes	Yes	Generalized skeletal under-mineralization Metaphyseal enlargement Disorganization on long bones	2 injections at 0.5 mg/kg: PTH increases	Day 15	3 mg/kg/day in 2 doses
Fisher 1 2015	*De novo*	M	None	At 11 month of age, global hypotonia, gross motor, fine motor and speech delays Dysphagia requiring gastrostomy tube feedings	Yes	N/A	Metaphyseal irregularities Diffuse osteopenia Short ribs with irregular rib ends Metaphyseal sclerosis at the ends of multiple long bones	Single dose of pamidronate (0.5 mg/kg IV): transient response but serum calcium rose to 13.8 mg/dl 2 weeks later	12 months	Ranged from 2.4 to 7.4 mg/kg per day
Fisher 2 2015	*De novo*	F	None	At day 26, failure of linear growth, poor weight gain, and cough	No	Yes	Multiple rib fractures Diffusely osteopenic bones with coarse trabecular markings diffuse symmetric periosteal reactions, and healing right fourth to ninth and left seventh to ninth lateral rib fractures	No	4 months	Ranged from 1.68 to2.7 mg/kg per day
Reh 2011	*De novo*	F	Oligoamnios and pregnancy-induced hypertension	At day 11, failing to thrive	No	No	Diffuse osteopenia with coarse trabecular changes in the long bones and thinning of the diaphyseal cortices but no fractures	Single dose of pamidronate (0.5 mg/kg iv) given at 2 weeks: 24 h normalized Ca but within 36 h became hypocalcemic	Day 23	20 mg/m^2^, PO twice-daily
Forman 2018	Assumed *de novo*	M	None	At day 3, respiratory distress, feeding difficulties, and depressed mental status	No	No	Diffuse demineralization and subperiosteal bone resorption, abnormal contour of the thoracic cage and metaphyseal irregularities in the long bones	Rejected due to concerns for prolonged hypocalcemia and possible respiratory distress in a patient with an ongoing oxygen requirement	Day 7	5 mg/kg/day
Gannon 2014	Paternal inheritance	M	Oligoamnios	At day 2, hypotonia, apnea and bradycardia	No	N/A	Diffuse demineralization, multiple rib fractures, chondrodystrophy of the distal humerus and femur, and a butterfly vertebra also noted on the chest radiograph	No	Before 21 days	9.6 mg/kg/day thrice daily
Obermannova 2009	*De novo* or paternal inheritance	M	none	At birth, respiratory distress leading to intubation and mechanical ventilation, narrowed thorax	Yes	N/A	Bell-shaped hypoplastic chest and visible leg fractures - multiple pathological skeletal fractures (ribs, right femur diaphysis, bilateral fractures of the proximal and distal right femur metaphyses) and diffuse skeletal under-mineralization with thin cortical layer of the long bones	Over three consecutive days at 0.5 mg/kg/d, transient suppression of serum calcium levels and PTH levels, subtotal then total parathyroidectomy at 8 weeks, but hyper-PTH and hypercalcemia three weeks later	No cinacalcet	N/A
Bai 1997	*De novo*	F	none	At 3 weeks, bone abnormalities (very soft skull, large fontanels, and wide open sutures, bowed femurs)	No	N/A	Diffuse osteopenia and fractures Severe generalized osteopenia and metaphyseal fractures of the proximal humeri and proximal and distal femur with periosteal calcification, marked impressiones digitatae of the frontal bones, lamina durae of the teeth demineralized, cortices of the long bones indistinct and split	N/A	No cinacalcet	N/A

Legend: F = female, M = male, PTH = parathyroid hormone, N/A = not applicable.

## 4 Discussion

In this paper, we describe a new case of NSHPT caused by the pathogenic heterozygous inactivating *CASR* variant Arg185Gln, with prenatal bone and renal features. As was the case in a few previous reports, treatment with cinacalcet successfully controlled hyperparathyroidism and corrected bone abnormalities in our patient. Table 1 summarizes the clinical presentation and management of NSHPT with the pathogenic heterozygous *CASR* variant Arg185Gln.

### 4.1 Prenatal features

To our knowledge, this is the first report of prenatal onset NSHPT with bone and renal presentation. In fact, children with NSHPT are often diagnosed during the first weeks of life as a result of poor feeding, polyuria, failure to thrive, hypotonia, and respiratory distress due to a poorly developed thoracic cage (Table 1). The only prenatal features that have been reported are oligohydramnios (Reh et al., 2011; Gannon et al., 2014) or, on the contrary, polyhydramnios (Murphy et al., 2016).

Interestingly, the association of short ribs and renal abnormality led to an initial diagnosis of Jeune syndrome in our patient. The same diagnosis was initially suspected in a male patient reported by Fisher et al. who presented at birth with global hypotonia, bell-shaped chest, and metaphyseal irregularities (Fisher et al., 2015). In this case, the patient was secondarily diagnosis with NSHPT at 11 months of age after further review of the radiographs revealed signs of metabolic bone disease (diffuse osteopenia, short ribs with irregular rib ends, and metaphyseal sclerosis at the ends of multiple long bones) and a biochemical evaluation indicated PTH-dependent hypercalcemia (Fisher et al., 2015). Jeune syndrome (MIM#208500) is an autosomal recessive skeletal ciliopathy in which a narrowed/bell-shaped thorax is associated with short ribs and irregular rib ends, short long bones with an irregular metaphysis, renal abnormalities, and less frequently, polydactyly, and hepatic, retinal, or pancreatic abnormalities (Baujat et al., 2013). Short ribs are noted if chest-to-abdominal circumference ratio is below 0.8, and a ratio below 0.6 is strongly suggestive of lethality (Yoshimura et al., 1996; Krakow et al., 2009). Our case report suggests that NSHPT can be considered in a differential diagnosis of Jeune syndrome pre- and postnatally. Prenatally, the family history and biochemical evaluation of the parents could help to differentiate these two disorders.

### 4.2 Genotype - phenotype correlation for the pathogenic variant Arg185Gln

Although NSHPT is usually caused by biallelic inactivation of the *CASR* gene, heterozygous *CASR* gene variants have also been implicated, notably the pathogenic variant Arg185Gln (Marx and Sinaii, 2020). The missense variant Arg185Gln is located in the extracellular domain of CASR that contains putative Ca^2+^-binding sites (Huang et al., 2009). *In vitro* studies have demonstrated the dominant negative inhibition of the wild-type *CASR* by this mutant (Marx and Sinaii, 2020; Obermannova et al., 2009). Interestingly, the pathogenic heterozygous variant Arg185Gln can also be found in FHH as was illustrated in the patient's father. The variability of severity (FHH *vs* NSHPT) with the same pathogenic heterozygous variant is not fully understood but may be at least partially due to paternal transmission (Fig. 2). Therefore, the more severe form (NSHPT) may occur with paternal or *de novo* transmission of the variant. In this situation, the fetus' abnormal CASR may have detected the normal maternal calcium level as low, leading to hyperparathyroidism (Reh et al., 2011). In such cases, fetal hyperparathyroidism often changes to the usual FHH phenotype at birth after separation from the maternal environment. Maternal vitamin D deficiency, which leads to a decrease in fetal CASR expression by a defect in CASR transactivation, may exacerbate fetal hyperparathyroidism (Zajickova et al., 2007). In our case, the mother had normal serum vitamin D levels when assessed after birth, but her status during pregnancy is unknown. In the literature, all seven NSHPT cases with the pathogenic heterozygous variant Arg185Gln were either *de novo* or paternally-transmitted (Table 1). At least fourteen individuals with the same variant were diagnosed with a FHH phenotype and no neonatal symptoms (Glaudo et al., 2016). The basis for this variability is not fully understood and may involve environmental factors and genetic modifiers.

### 4.3 Treatment

In NSHPT, hyperparathyroidism is considered to cause an increase in bone resorption. Consequently, the use of bisphosphonates which inhibit osteoclastic bone resorption seems logical. However, it has been reported that this treatment has variable efficacy in newborns, and may sometimes be accompanied by a rebound increase in serum PTH and hypercalcemia as was documented in our patient (Fisher et al., 2015; Reh et al., 2011; Sun et al., 2018; Murphy et al., 2016; Savas-Erdeve et al., 2016).

A better understanding of the molecular basis of NSHPT helps to define specific treatment such as calcimimetics. Within the transmembrane domain, cinacalcet binds to a separate site from the activating domain and changes CASR conformation (Sun et al., 2018; Capozza et al., 2018). This positive allosteric modulation enhances CASR sensitivity to extracellular calcium and specifically causes the calcium set point abnormality found in NSHPT patients (Garcia Soblechero et al., 2013). NSHPT patients with bi-allelic variants are usually unresponsive to cinacalcet (Marx and Sinaii, 2020). In contrast, as with our patient, several cases of NSHPT caused by the pathogenic heterozygous variant Arg185Gln have been successfully treated with cinacalcet (Fisher et al., 2015; Reh et al., 2011; Forman et al., 2019; Gannon et al., 2014) (Table 1), suggesting residual CASR functionality (Zhang et al., 2002). The dose of cinacalcet required to control hyperparathyroidism was highly variable among individuals (ranging from 2.4 to 9.6 mg/kg/day). As it has been previously reported (Fisher et al., 2015; Gannon et al., 2014), although cinacalcet normalizes serum PTH levels, it does not restore normal serum calcium levels. It has been suggested that this could be due to reduced sensitivity of diverse cells to extracellular calcium due to a CASR dominant-negative effect (Gannon et al., 2014).

These data reinforce the premise that medical management with cinacalcet can successfully control hypercalcemia in NSHPT caused by the pathogenic heterozygous variant Arg185Gln and prevents surgical treatment. Genetic diagnosis may contribute to the use of cinacalcet as a first-line treatment.

## 5 Conclusion

In this paper, we describe a case of NSHPT with prenatal onset of bone and renal features. Clinicians should be aware of this diagnosis in the presence of short ribs or a bell-shaped thoracic cage, and it should be borne in mind in the differential diagnosis when skeletal ciliopathies are suspected in the antenatal period. Phosphocalcic evaluation of both parents should be considered, as FHH is highly frequent, asymptomatic, and can be discovered at that time. It could guide the diagnosis of NSHPT. In addition, the mother's vitamin D status plays a role in the severity of NSHPT, and should be corrected in case of deficiency. Moreover, targeted detection of the recurrent pathogenic *CASR* variant Arg185Gln should be considered for similar antenatal presentations to confirm the diagnosis and to permit the initiation of cinacalcet as soon as possible, as efficacy in this variant has now been well-established.

## Funding

No financial assistance was received in support of the study.

## URLs

*ClinVar: https://www.ncbi.nlm.nih.gov/clinvar/

*NCBI Database: https://www.ncbi.nlm.nih.gov/

*Omim: https://www.omim.org/

## Declaration of competing interest

The authors declare that they have no known competing financial interests or personal relationships that could have appeared to influence the work reported in this paper.
